# Encoding the Sequence of Specific Autoantibodies Against beta-Amyloid and alpha-Synuclein in Neurodegenerative Diseases

**DOI:** 10.3389/fimmu.2019.02033

**Published:** 2019-08-27

**Authors:** Alexandra Albus, Marit Jördens, Moritz Möller, Richard Dodel

**Affiliations:** ^1^Chair of Geriatric Medicine, University Hospital Essen, University Duisburg-Essen, Essen, Germany; ^2^Department of Neurology, Philipps-University, Marburg, Germany

**Keywords:** B1 cell, naturally occurring autoantibodies, Alzheimer's disease, Parkinson's disease, single-cell RT-PCR, passive immunization strategy

## Abstract

There is no effective disease-modifying therapy for Alzheimer's or Parkinson's disease. As pathological hallmarks, the specific peptide amyloid-β and the specific protein α-Synuclein aggregate and deposit in and destabilize neurons, which lead to their degeneration. Within the context of a potential immunization strategy for these diseases, naturally occurring autoantibodies could play a crucial role in treatment due to their ability to inhibit peptide/protein aggregation and mediate their phagocytosis. We developed a procedure to extract the genetic information of such amyloid-β- and α-Synuclein- specific naturally occurring autoantibodies for future passive immunization strategies. We performed FACS-based single-cell sorting on whole blood donated from healthy individuals and performed single-cell RT-PCR analysis to amplify the coding sequences of antigen-binding regions of each antibody-secreting B1 cell. Sequences were further analyzed to determine CDR sequences and germline expression. Therefore, only low percentages of B1 cells obtained were amyloid-β^+^/α-Synuclein^+^. After cell sorting, the variable regions of full IgGs were sequenced, demonstrating preferred usage of IGVH3 and IGKV1. The study we present herein describes an approaching for extracting and amplifying the sequence information of autoantibodies based on single-cell analysis of donated blood and producing a recombinant antibody pool for potential passive immunization against neurodegenerative diseases. We sorted a small pool of CD20^+^ CD27^+^ CD43^+^ CD69^−^ IgG^+^ and Aβ^+^/α-Syn^+^ B cells.

## Introduction

Naturally occurring autoantibodies (nAbs) form a special group of immunoglobulins that are directed against self-components and form a part of the innate immune system. They are secreted by B1 cells, a distinctive subpopulation of B cells, and are generated early in development in the fetal liver (1). nAbs assume a special position among the pool of all antibodies because they are found in the sera of individuals even if there is no verifiable, previous contact with antigens or T cell participation (2, 3). Although they are established early in life, their maintenance in adults is enabled by self-replenishment (4, 5).

Recently, we and other groups identified nAbs that may play an important role in neuroprotection (6, 7). The two most frequent neurodegenerative diseases, Alzheimer's (AD) and Parkinson's (PD) disease, are characterized by misfolding, aggregation, and intra- or extracellular deposition of certain peptides and proteins (8). During AD progression, amyloid-β (Aβ) primarily deposits within the hippocampus (9). In the case of PD, intraneural deposits of proteins including α-Synuclein (α-Syn) as the main component are formed (10–12). Therefore, modifying the turnover of both peptide and protein by, for example, antibodies represent a promising approach for future therapies. The neuroprotective function of nAbs has been demonstrated by *in vitro* as well as in *in vivo* experiments. nAbs against Aβ (nAbs-Aβ) or α-Syn (nAbs-α-Syn) inhibit peptide/protein fibrillation and exhibit a rescue effect on microglial uptake (6, 13). Moreover, nAbs-Aβ application reduces Aβ toxicity and leads to an improvement in cognition in AD models (14). Furthermore, nAbs might exert a protective function because nAbs-Aβ titers are lower in AD patients than in age-matched controls (15, 16).

For clinical applications, two possibilities are conceivable. The first involves purification of nAbs from commercially available intravenous class G immunoglobulins (IVIg), which is already being used for a variety of neurological diseases, such as myasthenia gravis and multiple sclerosis (17, 18). However, IVIg is a limited and expensive resource, as its preparation is dependent on blood donations (19). A second possibility is recombinant production.

Based on the protective mechanisms of nAbs, we sought to develop a method for recombinant production of such antibodies for future therapeutic approaches because all of the previously conducted clinical trials have been unsuccessful and terminated. Surprisingly, clinical trials applying monoclonal antibodies against Aβ did not improve symptoms, potentially due to epitope specificity (20). Although a passive immunization approach for PD has been successfully tested in mice, no clinical trial with humans has been conducted (21).

To date, recombinant production of nAbs has not been reported. Sevigny et al. used an undefined pool of Aβ-recognizing memory B cells to isolate binding antibodies, similar to the experiments of Pascual et al., who performed comparable approaches for hyperphosphorylated tau (22, 23). Here, we extend this approach by focusing on Aβ/α-Syn-nAbs-producing B1 cell subpopulations and unraveling their genetic distinction.

## Methods

### Peptides

FITC-labeled Aβ (Bachem) was aggregated into amyloid-derived diffusible ligands (ADDLs) according to the protocol of Freir et al. (24). α-Syn (rPeptide) was used in monomeric form. Both were stored at −80°C until used in experiments. For verification experiments Aβ (Bachem) was additionally used in monomeric or oligomeric form. For monomers Aβ was dissolved in PBS to 1 mg/ml and stored at −80°C. For oligomers PBS dissolved Aβ with 1 mg/ml was incubated at 37°C for 24 h with constant agitation and then stored at −80°C.

### Fluorescein-Labeling of α-Syn

α-Syn was diluted to 20 mg/ml in PBS. The solution obtained was labeled according to the manufacturer's protocol with the Lightning-Link®-Fluorescein labeling kit (Innova Bioscience). Briefly, 1 μl of the included LL-modifier was mixed with the protein. This mixture was then resuspended with lyophilized Lightning-Link®. According to the manufacturer, 100% of the protein was FITC labeled. FITC-labeled α-Syn was stored at −20°C for a short time or at −80°C for longer storage.

### Blood Samples and Donors

The study was approved by the Ethics Committee of the Philipps-University Marburg (no: 172/15) and conducted in accordance with the Declaration of Helsinki (25). Ten healthy donors between 20 and 30 years of age were included. After providing written informed consent, blood samples were collected by standard peripheral vein-puncture into 9-ml EDTA S-monovettes (Sarstedt AG & Co.) and processed instantly.

### Isolation of PBMCs

Initially, 20 ml venous EDTA blood was diluted with 20 ml PBS. In a new tube, 10 ml of Biocoll® (Merck KGaA) was gently overlaid with the blood dilution without mixing the two phases. This preparation was centrifuged at 1,500 rpm for 30 min at 20°C without braking. The PBMC fraction, which was turbid and layered below the top fraction, was carefully transferred into a new 50-ml tube, washed twice with 40 ml PBS supplemented with 1 mM EDTA (Sigma-Aldrich® Corporation), and again centrifuged at 1,500 rpm for 20 min at 20°C with braking. The supernatant was discarded, and the pellet was resuspended in FACS buffer [PBS + 0.4 % fetal calf serum (FCS)].

### B Cell Enrichment

The obtained suspension of PBMCs contained lymphocytes, natural killer cells, macrophages, and monocytes. To separate B lymphocytes from the other PBMCs, the EasySep^TM^ human B cell enrichment kit without CD43 depletion (STEMCELL Technologies Inc.) was used at room temperature following the manufacturer's instructions. The advantage of this negative selection method was that the desired B1 cells remained untouched during the process and thus were not activated.

### Staining and Fluorophores

The enriched B cells were washed by centrifugation twice with FACS buffer for 5 min at 4°C and 1,200 rpm; the supernatant was discarded, and the pellet was resuspended in 300 μl FACS buffer. Afterwards, the concentration of B cells was determined using a Neubauer counting chamber (Brand GmbH & Co. KG). Next, the cell suspension was split into different FACS tubes (Sarstedt) on ice and centrifuged at 1,200 rpm and 4°C for 5 min. The supernatant was discarded, and the pellet was resuspended in 200 μl PBS with 10 % FCS. This suspension of purified B cells was incubated on ice and in the dark for 20 min with 300 nM Aβ-FITC or α-Syn-FITC to select cells expressing antibodies directed against Aβ or α-Syn on their cell surface. Afterwards, the cells were washed for 5 min at 1,200 rpm and 4°C.

For the next step, the cells were stained for 30 min on ice and in the dark with antibodies against a selection of CD markers that are expressed on viable B1 cells. To differentiate B1 cells from the other cells in the suspension, antibodies against CD20, CD27, CD43, CD69, and IgG were used (26). Distinct CD markers can be distinguished based on antibodies with individual fluorophores. All fluorophore-coupled CD markers and the IgG marker were purchased from BD Biosciences.

### Single-Cell Sorting

B1 cells (CD20^+^, CD27^+^, CD43^+^, and CD69^−^) presenting IgG^+^ B cell receptors against Aβ/α-Syn on their surface were detected using the cell sorter MoFlo Astrios (Beckman Coulter). Sorting was carried out in round-bottom 96-well PCR plates without a skirt (Sarstedt) containing 4 μl/well of sorting buffer (0.5-fold PBS containing 10 mM DTT (Invitrogen), 8 U RNasin (Promega), and 0.4 U 5′-3′ Stop RNase Inhibitor [5 Prime GmbH) (27)]. After sorting, the plates were placed immediately on dry ice and stored at −80°C.

### Single-Cell RT-PCR and Ig Gene Amplification

To determine the sequences of the antigen-binding domain of nAbs against Aβ/-α-Syn, single-cell mRNA was isolated first, after which the mRNA was transcribed into cDNA by reverse transcription (RT) (26). A PCR-based amplification followed according to Tiller et al., with minor adjustments (27). The RT reaction for each cell was performed in a total volume of 16 μl/well, consisting of nuclease-free water (Qiagen), 1 μl of 0.1 M DTT (Invitrogen); 150 ng random hexamer primer [pd(N)6, GE Healthcare], 0.5 μl dNTPs (25 mM each) (Thermo Scientific), 0.5% v/v Igepal CA-630 (Sigma-Aldrich), 6 U 5 Prime™ Stop RNAse Inhibitor (Fisher Scientific), 4 U RNasin (Promega), RT-buffer (Invitrogen), and 50 U Superscript III reverse transcriptase (Invitrogen). This master mix was added to each well, and the plate was sealed with optically clear sealing tape (Sarstedt). RT was performed using a thermal cycler (MyCycler, Bio-Rad Laboratories) with the following steps: 42°C for 10 min, 25°C for 10 min, 50°C for 60 min, and 94°C for 5 min. At the end of the reaction, the plate was cooled to 4°C before storage at −20°C. As the nAbs are subtype G immunoglobulins, each consists of two heavy chains (IgH) as well as two light chains. These are interconnected via disulfide bridges; thereby the light chains are of kappa (Igκ) or lambda (Igλ) subtype. All the cells were checked for both light chains because an IgG always comprises two light chains of the same type. IgH, Igκ, and Igλ, as well as β-actin (positive control) gene transcripts, were amplified separately. Hence, the RT product was split into four equal parts for the subsequent PCR steps. All PCR reactions were performed in PCR tubes (Sapphire PCR 8-tube strips, 0.2 ml, Greiner Bio-One International GmbH) in a total volume of 40 μl per tube. Each tube contained 3.5 μl cDNA, nuclease-free water (Qiagen), 300 μM dNTPs, 25 mM each (Thermo Scientific), 200 nM specific primer/primer-mix as listed in Supplemental Table 1 (microSynth), Q-Solution (Qiagen), PCR-buffer (Qiagen), 1 mM MgCl (Qiagen), and 1.2 U HotStar Taq DNA Polymerase (Qiagen). For β-actin (positive control), the primer concentration was adjusted to 400 nM. The PCR protocols were the same for IgH and Igκ but were different for Igλ and β-actin. The detailed protocols can be found as an additional file (see Supplemental Table 1) and were performed using a thermal cycler (PTC-200 thermal cycler, MJ Research Inc.). Secondary PCR was then carried out. The master mixes were mostly the same, only the primers were adjusted. The PCR protocols were also adjusted for the secondary primers, and cycles were reduced. Detailed protocols can be found in Supplemental Table 2.

### Agarose Gel Analysis of PCR Products

1.5% Agarose (Biozym Scientific GmbH) gels were prepared in TBE buffer, and SYBR®-gold (Thermo Fisher Scientific) was added. The PCR samples were mixed with blue dye (PeqLab Biotechnologie GmbH) before loading, and a 100-base pair DNA ladder (Invitrogen) was used. Electrophoresis was performed for 90 min at 110 mV in TBE as a running buffer, and the PCR products were evaluated under UV light. The PCR products were purified after amplification (Gene Matrix PCR Clean up, roboklon GmbH) following the manufacturer's instructions.

### Sequencing and Analysis

Sequencing was performed by Seqlab Sequence Laboratories GmbH. The sequences obtained were reviewed and compared to each other and checked by sequence analysis at vbase2.org (28). This website allows analysis of complementarity determining regions (CDRs), and several sequences can compared with each other with regard to CDR similarity. This comparison of sequence patterns allows for determination of the antigen-binding domain of the antibody. Due to the patenting process, the sequences are not provided in the Results section.

### Further Verification of Cloned Antibodies

Three antibody constructs were cloned successfully into IgG1 backbones and transfected into HEK293 cells by industrial provider (Yumab GmbH). One of these constructs was additionally cloned as single chain antibody scFv-Fc (Yumab GmbH). All antibodies were further collected from the cell culture supernatant and were purified using protein A.

#### Aβ ELISA

The recombinant antibodies were tested in their binding ability to monomeric and oligomeric Aβ as well as to ADDLs. 96-well round-bottom high-binding ELISA plates (Sarstedt) were coated with 5 μg/ml peptide (in PBS) over night at 4°C. Next day, plates were washed three times with washing buffer (PBS + 0.05% Tween-20) using a plate washer (Amersham Biotrak II Plate Washer, GE Healthcare) and blocked with blocking buffer (Roti + 0.1% Tween-20; Roti-block, Roth; Tween-20, Applichem GmbH) for 1 h at 37°C. Next to the recombinant antibodies B06-IgG, B07-IgG, B07-scFvFc, and C06-IgG a commercially available IVIg preparation (10% octagam, Octapharma) was used; from this IVIg preparation nAbs-Aβ were purified (29). Samples were diluted in blocking buffer to a final concentration of 10 μg/ml (except for B07-scFvFc and nAbs-Aβ with 1.25 μg/ml due to high signal) and applied in duplets and incubated for 1 h at room temperature using constant agitation. Plates were washed again three times with washing buffer and incubated with secondary antibody for 1 h at RT under constant agitation. As secondary antibody HRP-conjugated anti-human antibody (Calbiochem) was used in 1:2,000 in blocking buffer. Plates were washed for three times with washing buffer and incubated for 20 min with TMB (Merck). The reaction was stopped with sulfuric acid (5% H_2_SO_4_, Sigma-Aldrich). Signals were measured at 450 nm with a plate reader (Tecan Infinite M200), and the background signal was subtracted.

### Statistical Analysis

If not stated otherwise, the results are presented as the mean ± standard deviation. The IGV gene distribution is shown as percentages of all analyzed sequences.

## Results

### Qualitative Selection of nAb-Secreting B1 Cells; nAb-Secreting B1 Cells Are a Small and Distinct Subtype Among B Cells

**Figure 1:**
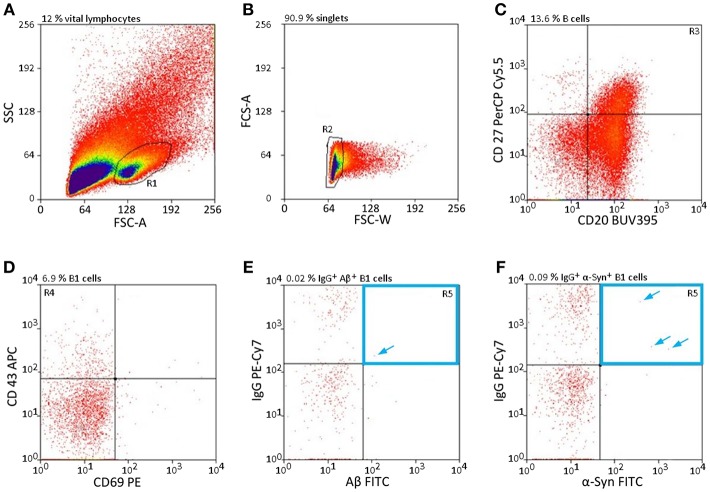
nAb-secreting B1 cells are rare and highly specific. **(A–F)** Gating strategy to sort only CD20^+^ CD27^+^ CD43^+^ CD69^−^ IgG^+^, and Aβ^+^ **(E)** /α-Syn^+^ **(F)** B cells. Percentages above each plot indicate the proportion of cells resulting from the previous gate. Arrows indicate single cells in gate R5. CD, cluster of differentiation; FSC, forward scatter; SSC, sideward scatter.

The cell gating was qualitatively assessed by FACS single-cell sorting. Approximately 30% of the leukocytes are lymphocytes; of those, 23% are allotted to B cells (30), which again can be divided into three subclasses: B1 cells, follicular B2 cells, and marginal zone B cells (3). As B1 cells are a subgroup of B cells, they are even rarer in the peripheral blood, especially those that secrete specific nAbs subclasses such as nAbs-Aβ and nAbs-α-Syn. To obtain specific sorting of these limited number of cells, a defined setting had to be chosen. Figure 1 shows the selection criteria to overcome the limitation of these low levels of lymphocytes (Figure 1). Only a small subpopulation (0.0063 ± 0.0042% for Aβ, 0.0087 ± 0.0014 for α-Syn) of all vital lymphocytes was scored positively (Figures 1E,F).

To determine whether the newly established protocol for the isolation of nAb-Aβ/α-Syn-producing B1 cells provides reproducible results, replications were performed with blood samples from different individuals. The numbers of nAb-Aβ- or nAb-α-Syn-secreting B1 cells were rather stable among test replications (Table 1).

**Table 1:** Percentages of Aβ^+^/α-Syn^+^ and IgG^+^ B1 cells demonstrate their limited appearance.

	**B cells**	**B1 cells**	**IgG^**+**^ B1 cells**	**Aβ^+^ and IgG^**+**^ B1 cells**	**α-Syn^**+**^ and IgG^**+**^ B1 cells**
Mean percentage from the B cell-population	100%	1.69% (±0.6%)	0.29% (±0.11%)	0.0063% (±0.0042%)	0.0087% (±0.0014%)
Mean percentage from the B1 cell-population		100%	17.04% (±6.73%)	0.37% (±0.22%)	0.51% (±0.082%)

*Fractions of Aβ^+^/α-Syn^+^ B1 cells were calculated on the basis of 10 measurements. Means ± SDs are shown*.

The blood of each donor was divided into two samples, which were examined separately by FACS. The respective mean of both measurements is shown in Table 1. The mean of all measurements was calculated, as well as the percentages of the categories of the entire B cell population. Over this, the percentages of B1 cell categories and the respective standard deviations were stated.

**Figure 2:**
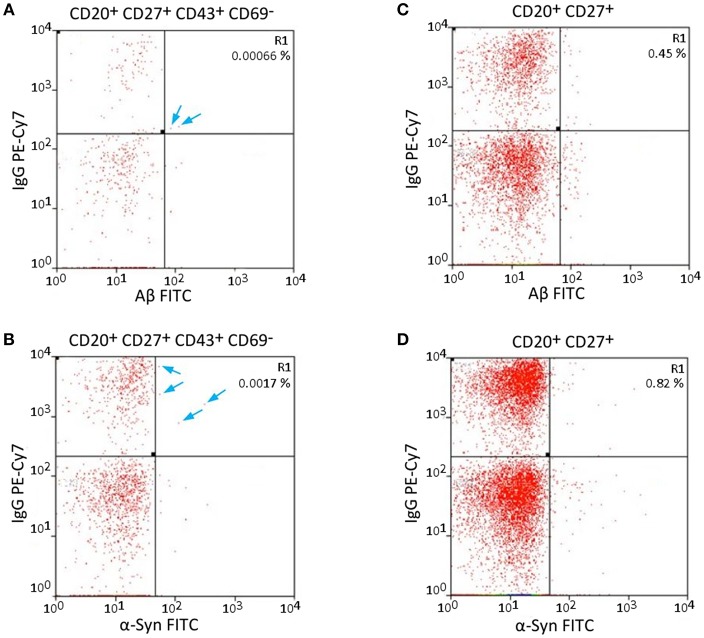
Specific and non-specific gating of Aβ- and α-Syn-FITC signals shows possible error sources. **(A,C)** Aβ-FITC and **(B,D)** α-Syn-FITC. Different gates were applied to the same sample. **(A,B)** Same gating procedure as in Figure 1. **(C,D)** CD43 and CD69 were omitted for gating. The cells in gate R1 are not necessarily B1 cells, as the gating is not B1 cell specific. Arrows indicate single cells in gate R1 in **(A,B)**.

The specificity of Aβ/α-Syn binding can be validated by the results of FACS measurements. Aβ should only bind to nAb-Aβ-producing B1 cells and α-Syn only to nAb-α-Syn-producing B1 cells. Therefore, dot plots of the Aβ-FITC signal and the IgG-PE-CY7 signal/α-Syn-FITC signal and the IgG-PE-CY7 signal should appear somewhat similar when the gating is changed. Figure 2 depicts the comparison of the Aβ-FITC/α-Syn-FITC signal with B1 cell-specific and non-specific gating. In Figure 2A (Aβ) and Figure 2B (α-Syn), the gating procedure was performed as shown in Figure 1, whereas in Figure 2C (Aβ) and Figure 2D (α-Syn), only the non-specific gates R1, R2, and R3 (Figures 1A–C) were applied. If the binding is specific, the cell numbers in gate R1 of Figure 2 should remain constant. However, gate R1 in Figure 2A contained two cells for Aβ and in Figure 2B 4 cells for α-Syn, and gate R1 in Figure 2B contained 19 cells and in Figure 2D 34 cells. Accordingly, the number of cells in gate R1 was 9.5 (Aβ)/5.7 (α-Syn) times higher in the case of B1 cell-non-specific gating (CD43 and CD69 markers were omitted). However, if the binding of Aβ/α-Syn to the cells is completely non-specific, the cell number in gate R1 of Figures 2B,D should be ~70 (Aβ)/50 (α-Syn) times higher, as B1 cells account for 1.69% of the entire B cell population (Table 1). This result indicates that the binding of Aβ/α-Syn to the cells originates from specific interactions with the B cell receptor on the surface of nAb-Aβ/α-Syn-producing B1 cells.

### RT-PCR

**Figure 3:**
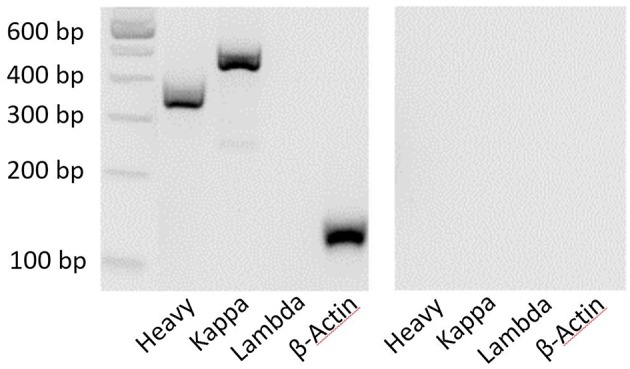
Clear bands by agarose gel electrophoresis show the composition of antibody chains. PCR products were electrophoresed for 90 min at 110 mV. The 100-bp DNA ladder provides an objective size indicator. A representative sample is shown on the left, and the negative control (water) is shown on the right.

Each sorted nAb belongs to the IgG type and consists of two heavy chains (IgH) and two light chains of the same subtype (Igκ or Igλ) (31). The success of the described single-cell sorting and RT-PCR approach was visualized by agarose gel electrophoresis. Ideally, one clear band for an antibody heavy chain and one band for one type of light chain would be detected. In addition, β-actin was used as a positive control to demonstrate the overall presence of cDNA in the samples (Figure 3). Distinct PCR master mixes were checked for impurities by performing negative controls (with nuclease free water) in each PCR series.

### Phylogenetic Analysis of Heavy and Light Chains

**Figure 4:**
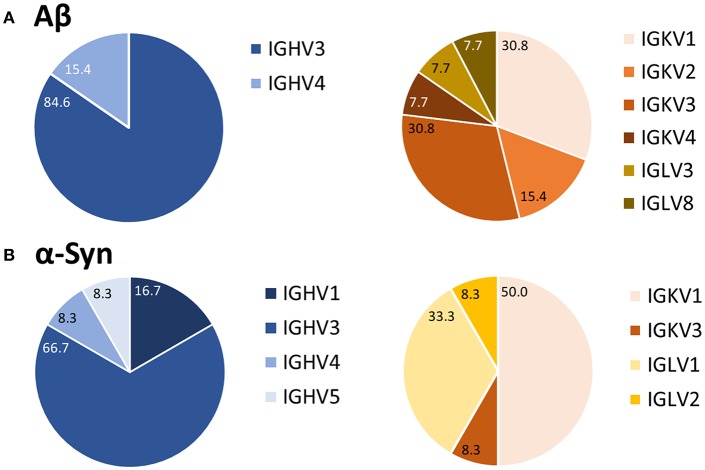
Preferred usage of IGVH3 and IGKV1 of Aβ **(A)** and α-Syn **(B)** variable antibody regions. After sequence analysis, the usages of germline genes were compared using the vbase2.org tool (31). Percentages of each variable region family are marked. For Aβ, 13 antibodies were analyzed; 12 were analyzed for α-Syn.

After purification of the PCR products and sequence analysis, germline expression of variable heavy (IGVH) and light (IGKV/IGLV) chains was determined (Figure 4). Depending on the type of primer used, one could determine to which of the five IGVH families the IGVH of interest belonged (see Supplemental Table 1). Such comparison revealed a preferred usage of IGVH3 for the heavy chain for both Aβ (85%) and α-Syn (67%) antibodies. In the case of light chains, the kappa chain (IGKV) belongs to one of four different IGKV families, and the lambda chain (IGLV) belongs to one of eight different IGLV families (see Supplemental Table 1). IGKV1, a kappa chain subtype, appeared to be preferred for Aβ and α-Syn (Aβ 31%, α-Syn 50%). However, other germline usages of light chains differed for the Aβ and α-Syn antibody sequences.

### Verification of Binding

**Figure 5:**
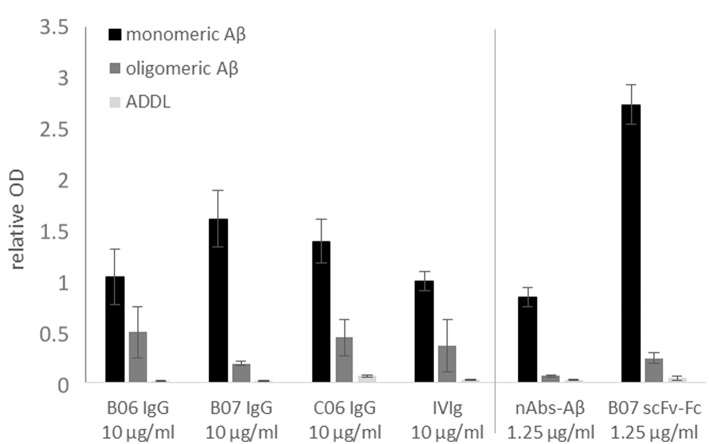
Binding verification of cloned antibodies by ELISA. Monomeric or oligomeric Aβ as well as ADDLs were coated with 5 μg/ml onto the surface of an ELISA plate. Antibodies were applied with 10 or 1.25 μg/ml (nAbs-Aβ and B07 scFv-Fc) and visualized by HRP-conjugated secondary anti-human antibody. The data were normalized to IVIg binding on monomeric Aβ. Mean values ± SD of three independent experiments are shown. B06 IgG, B07 IgG, C06 IgG, and B07 scFv-Fc mentioned different antibody clones.

To verify the successful sequencing and cloning, each antibody had to be checked for antigen-specific binding. The antigen-binding of first cloned antibodies against Aβ was tested using ELISA. Next to monomeric Aβ aggregated Aβ and ADDLs were tested (Figure 5). All cloned antibodies (B06, B07, and C06) showed a distinct and specific binding to monomeric Aβ. The single chain construct antibody B07 scFv-Fc had such a strong binding in comparison to the others that it was further diluted (1.25 μg/ml instead of 10 μg/ml). IVIg (as 10 μg/ml) and nAbs-Aβ (as 1.25 μg/ml) were used as references. Results were set in relation to the IVIg signal on monomers. The binding to aggregated Aβ and ADDLs was small for all antibodies.

## Discussion

In this study we provide a feasible and valid method to obtaining the coding sequences of nAbs. The development of recombinant antibodies requires several checkpoints due to many possible causes of failure. In the first step, a gating panel for isolating distinct B-lymphocytes must be selected. In our setting, we used an already-established gating panel for B1 cells consisting of CD20^+^, CD27^+^, CD43^+^, CD69^−^, and IgG^+^ (32). However, factors defining B1 cells are still under debate (32–35). For example, Griffin et al. termed B1 cells according to the CD marker panel, others have hypothesized that these cells are more likely to be plasmablasts or mischaracterized due to T cell doublets. Thus, the proposed markers are not sufficient for characterization. Descatoire et al. (33) and Perez-Andres et al. (34) reported some challenges to excluding false-positive cells, such as doublet gating and pre-enrichment of CD19^+^ cells. In our study, we circumvented this issue by performing pre-enrichment of B cells by removing macrophages, monocytes, T cells, and natural killer cells with a B cell enrichment kit. Furthermore, we excluded B:T doublets by our gating scheme, similar to Griffin et al. (Figure 1B). Any remaining activated T cells were excluded by negative CD69 gating (Figure 1D). In addition, we recorded CD20^+^, CD27^+^ and CD43^+^ cells as a small subset of total B cells, as noted by Descatoire et al. (33). Therefore, we applied the B1 cell gating scheme from Griffin et al., and we agree that experiments with B1 cells require accurate exclusion of non-target cells. Nonetheless, we performed no additional live/dead staining to exclude dead cells from the analysis due to the wide range of fluorophores used, though we did exclude most dead cells by the first SSC-FSC gating. In prior HOECHST experiments, we were able to exclude the majority of dead cells (Supplemental Figure 1). As possible non-specific FITC signal was intensively investigated in preliminary experiments, we used different percentages of FCS and albumin to block non-specific binding (Supplemental Figure 2), with 10% FCS showing the best results. Although additional experiments with unlabeled peptide/protein blocking were missing and represent a limitation of this study specific binding of cloned antibodies demonstrate a distinct interaction with the peptide/protein. To verify successful sorting and PCR amplification of Aβ^+^ and α-Syn^+^ B1 cells, the variable regions of the analyzed antibodies were transfected into IgG1 constructs and several of the antibodies were cloned. Therefore, first binding assays were performed to test the successful sorting, amplification and cloning of the antibodies against Aβ. The four different antibodies showed different binding intensities to the different Aβ conformation states (Figure 5). Thereby, the single chain construct B07 scFv-Fc bound strongest to Aβ. The other cloned antibodies showed stronger bindings compared to IVIg and therefore demonstrated a successful verification of prior assays. However, ELISA results showed higher SD in Aβ oligo binding (Figure 5). Due to oligomer preparation it is difficult to enable exactly the same aggregation state. Thus, each ELISA could be coated with different oligomeric peptide, which leads to differences in the available paratopes for the antibodies. The ADDL binding was nearly undetectable. Although we used ADDL for the antibody sorting the verification of the ADDL binding was not successful. Nevertheless, the investigated recombinant antibodies bind to monomeric Aβ and in a decreased pattern to oligomeric Aβ in ELISA studies.

Regarding the gating scheme for B1 cells during FACS experiments, the use of the appropriate Aβ preparation is a highly discussed topic. As it is known that nAbs-Aβ preferably bind to oligomeric forms of Aβ (36), we used ADDLs for Aβ^+^ B1 cell sorting. Because of the complicated binding method used to maintain the oligomeric state, the protocol of Freir et al. resulted in a highly robust conformation of Aβ oligomers (24). We used the protocol for these stable oligomeric ADDL forms based on this binding characteristic, which we could reproduce in our setting with FITC-labeled Aβ (Supplemental Figure 3).

The findings of this study are consistent with those of Pascual et al. (22). As already shown in their study about naturally anti-Tau antibodies the usage of IGHV3 family seemed to be higher for autoantigen reactive antibodies (21). With 35% for naïve B cells the general usage of IGHV family is distributed more equally (37). The B1 cells, which were investigated in this study, have the same origin as other B1 cell derived antibodies. Thereby, this could indicate a common origin of all naturally occurring autoantibodies. Further, the usage of IGKV seems to be preferred for B1 cell derived autoantibodies (21), although we also identified IGLV usage for our analyzed antibodies. In comparison, the general ratio between kappa and lambda chain occurrence amounts to 60:40 (38). These findings may indicate a common origin of all naturally occurring autoantibodies.

## Conclusion

In conclusion, we present a workflow to identify the coding sequences of antibodies using naturally occurring autoantibodies as an example. The identified antibody sequences will be examined for potential posttranslational modifications to verify the ability to form a functional antibody for testing *in vitro* and *in vivo*. This approach will lead to the generation of monoclonal antibodies based on naturally occurring autoantibodies, which can be used for a passive immunization strategy. Furthermore, this approach is possibly transferable to other autoimmune diseases, which to date are treated with IVIg. Thereby, their treatment might be independent of IVIg preparations (17, 18).

## Data Availability

The datasets for this manuscript are not publicly available because they are confidential. However, the datasets used and/or analyzed during the current study are available from the corresponding author on reasonable request. Requests to access the datasets should be directed to Richard.Dodel@uk-essen.de.

## Ethics Statement

This study was carried out in accordance with the recommendations of the Ethics Committee of the Philipps-University Marburg with written informed consent from all subjects. All subjects gave written informed consent in accordance with the Declaration of Helsinki. The protocol was approved by the Ethics Committee of the Philipps-University Marburg (no: 172/15).

## Author Contributions

AA and RD conceptualized and designed the study. AA, MJ, and MM performed the experiments and analyzed the data. AA and MJ drafted the initial manuscript. AA, MJ, MM, and RD reviewed and revised the manuscript. All authors approved the final manuscript as submitted and agreed to be accountable for all aspects of the work.

### Conflict of Interest Statement

The authors declare that the research was conducted in the absence of any commercial or financial relationships that could be construed as a potential conflict of interest.

## Supplementary Material

Supplemental Figure 1 HOECHST staining of lymphocytes **(A)** and other components **(B)**. **(A)** HOECHST staining was negative for 99.24% of gated lymphocytes (indicated by a blue arrow). **(B)** Focusing on more abundant components, only 7.32% of cells show HOECHST negative staining.

Supplemental Figure 2 Different approaches to block non-specific Aβ binding. During Aβ-FITC staining of B cells, different blocking agents were tested. Next, 1% albumin, 0.4% FCS, and 10% FCS were used as blocking solutions.

Supplemental Figure 3 Large Aβ-oligomers formed by the Freir protocol (23) shown by size exclusion chromatography. The first peak shows molecules of ~82 kDa. Because the shape of the peak is asymmetric, it is likely that it represents Aβ oligomers of different sizes. The second peak shows molecules of ~9.7 kDa, which corresponds to the molecular weight of Aβ-dimers. The last peak represents the solvent PBS.

Supplemental Table 1 Primer sets for each Ig chain PCR.

Supplemental Table 2 PCR protocols.
