# Supplementary figures and images for: Encoding the Sequence of Specific Autoantibodies Against beta-Amyloid and alpha-Synuclein in Neurodegenerative Diseases
Source: Front Immunol. 2019 Aug 27;10:2033. doi: 10.3389/fimmu.2019.02033 (PMC6718452; doi:10.3389/fimmu.2019.02033)

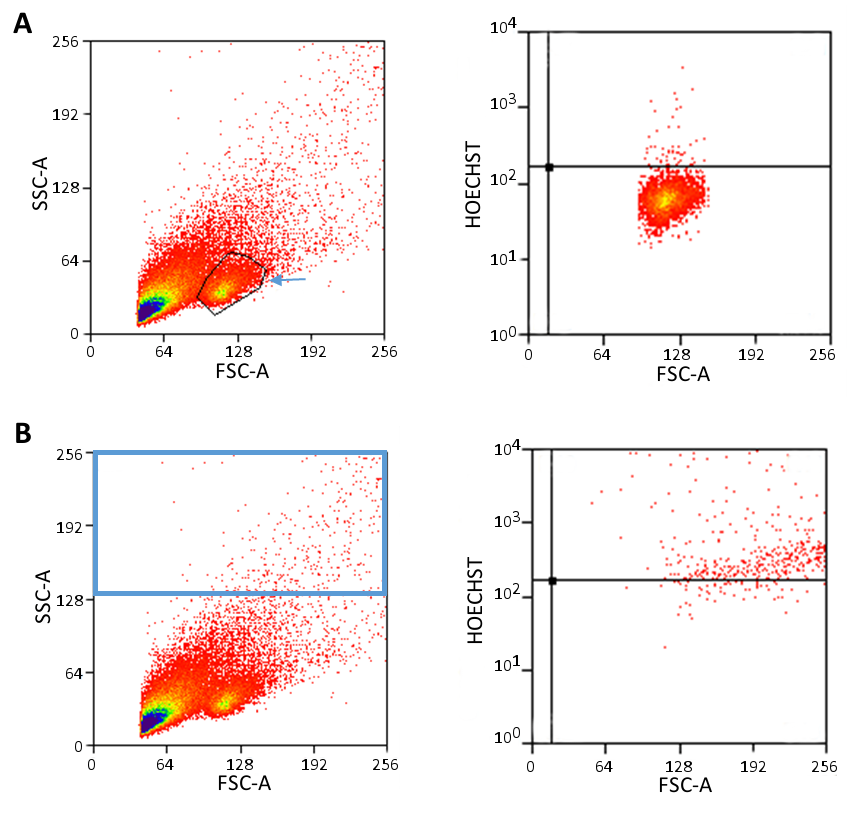

Supplement: Supplemental Figure 1 — HOECHST staining of lymphocytes (A) and other components (B). (A) HOECHST staining was negative for 99.24% of gated lymphocytes (indicated by a blue arrow). (B) Focusing on more abundant components, only 7.32% of cells show HOECHST negative staining. [file Image_1.TIF]

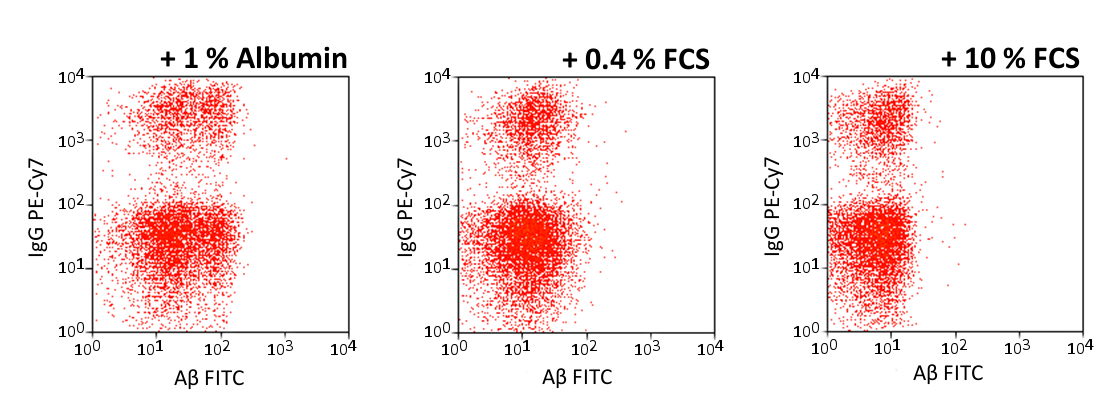

Supplement: Supplemental Figure 2 — Different approaches to block non-specific Aβ binding. During Aβ-FITC staining of B cells, different blocking agents were tested. Next, 1% albumin, 0.4% FCS, and 10% FCS were used as blocking solutions. [file Image_2.TIF]

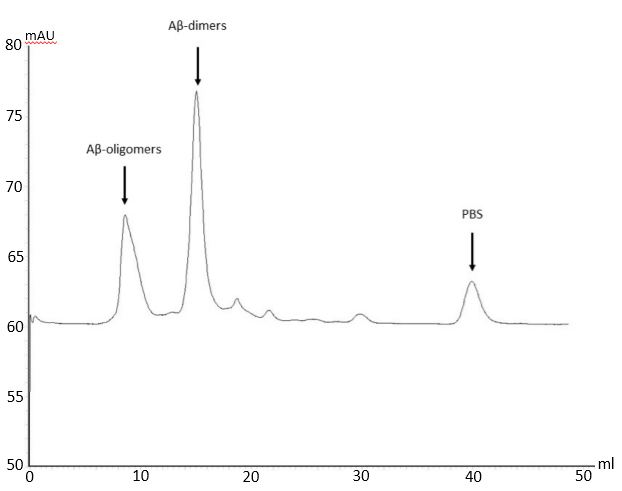

Supplement: Supplemental Figure 3 — Large Aβ-oligomers formed by the Freir protocol (23) shown by size exclusion chromatography. The first peak shows molecules of ~82 kDa. Because the shape of the peak is asymmetric, it is likely that it represents Aβ oligomers of different sizes. The second peak shows molecules of ~9.7 kDa, which corresponds to the molecular weight of Aβ-dimers. The last peak represents the solvent PBS. [file Image_3.TIF]
