# Supplementary material for: Encoding the Sequence of Specific Autoantibodies Against beta-Amyloid and alpha-Synuclein in Neurodegenerative Diseases
Source: Front Immunol. 2019 Aug 27;10:2033. doi: 10.3389/fimmu.2019.02033 (PMC6718452; doi:10.3389/fimmu.2019.02033)
Supplement: Supplemental Table 1 — Primer sets for each Ig chain PCR. [file Table_1.DOCX]

**supplemental data**

**Supplemental table 1: Primer sets for each Ig chain PCR**

| **Heavy** | **Forward primer** | | **Reverse primer** | |
| --- | --- | --- | --- | --- |
| First PCR | 5’ L-VH1 | ACAGGTGCCCACTCCCAGGTGCAG | 3’ Cγ CH1 | GGAAGGTGTGCACGCCGCTGG TC |
|  | 5’ L-VH3 | AAGGTGTCCAGTGTGARGTGCAG | 3’ Cµ CH1 | GGGAATTCTCACAGGAGACGA |
|  | 5’ L-VH4/6 | CCCAGATGGGTCCTGTCCCAGGTGCAG |  |  |
|  | 5’ L-VH5 | CAAGGAGTCTGTTCCGAGGTGCAG |  |  |
| Second PCR | 5’ Age VH1/5 | CTGCAACCGGTGTACATTCCGAGGTGCAGCTGGTGCAG | 3’ Sal JH1/2 | TGCGAAGTCGACGCCTGAGGAGACGGTGACCAG |
|  | 5’ Age VH3 | CTGCAACCGGTGTACATTCTGAGGTGCAGCTGGTGGAG | 3’ Sal JH3 | TGCGAAGTCGACGCTGAAGAGACGGTGACCATTG |
|  | 5’ Age VH4 | CTGCAACCGGTGTACATTCCCAGGTGCAGCTGCAGGAG | 3’ Sal JH4/5 | TGCGAAGTCGACGCTGAGGAGACGTGACCAG |
|  | 5’ Age VH3-23 | CTGCAACCGGTGTACATTCTGAGGTGCAGCTGTTGGAG | 3’ Sal JH6 | TGCGAAGTCGACGCTGAGGAGACGGTGACCGTG |
|  | 5’ Age VH4-34 | CTGCAACCGGTGTACATTCCCAGGTGCAGCTACAGCAGTG |  |  |
| **Kappa** | **Forward primer** | | **Reverse primer** | |
| First PCR | 5’ LVκ1/2 | ATGAGGSTCCCYGCTCAGCTGCTGG | 3’ Cκ 543 | GTTTCTCGTAGTCTGCTTTGCTCA |
|  | 5’ LVκ3 | CTCTTCCTCCTGCTACTCTGGCTCCCAG |  |  |
|  | 5’ LVκ4 | ATTTCTCTGTTGCTCTGGATCTCTG |  |  |
| Second PCR | 5’ Pan Vκ | ATGACCCAGWCTCCABYCWCCCTG | 3’ Cκ 494 | GTGCTGTCCTTGCTGTCCTGCT |
| **Lambda** | **Forward primer** | | **Reverse primer** | |
| First PCR | 5’ L Vλ1 | GGTCCTGGGCCCAGTCTGTGCTG | 3’ Cλ | CACCAGTGTGGCCTTGTTGCTTG |
|  | 5’ L Vλ2 | GGTCCTGGGCCCAGTCTGCCCTG |  |  |
|  | 5’ L Vλ3 | GCTCTGTGACCTCCTATGAGCTG |  |  |
|  | 5’ L Vλ4/5 | GGTCTCTCTCSCAGCYTGTGCTG |  |  |
|  | 5’ L Vλ6 | GTTCTTGGGCCAATTTTATGCTG |  |  |
|  | 5’ L Vλ7 | GGTCCAATTCYCAGGCTGTGGTG |  |  |
|  | 5’ L Vλ8 | GAGTGGATTCTCAGACTGTGGTG |  |  |
| Second PCR | 5’ Age Vλ1 | CTGCTACCGGTTCCTGGGCCCAGTCTGTGCTGACKCAG | 3’ XhoI Cλ | CTCCTCACTCGAGGGYGGGAACAGAGTG |
|  | 5’ Age Vλ2 | CTGCTACCGGTTCCTGGGCCCAGTCTGCCCTGACTCAG |  |  |
|  | 5’ Age Vλ3 | CTGCTACCGGTTCTGTGACCTCCTATGAGCTGACWCAG |  |  |
|  | 5’ Age Vλ4/5 | CTGCTACCGGTTCTCTCTCSCAGCYTGTGCTGACTCA |  |  |
|  | 5’ Age Vλ6 | CTGCTACCGGTTCTTGGGCCAATTTTATGCTGACTCAG |  |  |
|  | 5’ Age Vλ7/8 | CTGCTACCGGTTCCAATTCYCAGRCTGTGGTGACYCAG |  |  |
| **β-actin** | **Forward primer** | | **Reverse primer** | |
| First PCR | FP 3 | CTCCTCCCTGGAGAAGAGCTAC | RP 3 | TGAAGGTAGTTTCGTGGATGC |

**Supplemental table 2: PCR protocols**

| Heavy- and Kappa-chain reactions | | | | | | | | | | |
| --- | --- | --- | --- | --- | --- | --- | --- | --- | --- | --- |
| First PCR | | | | | Second PCR | | | | | |
| 15 minutes | 95 °C | |  | | 15 minutes | | | 95 °C | |  |
| 30 seconds | 94 °C | | 40 x | | 30 seconds | | | 94 °C | | 30 x |
| 30 seconds | 58 °C | |  |  | 30 seconds | | | 58 °C | |  |
| 55 seconds | 72 °C | |  |  | 45 seconds | | | 72 °C | |  |
| 10 minutes | 72 °C | |  | | 10 minutes | | | 72 °C | |  |
| ∞ | 4 °C | |  | | ∞ | | | 4 °C | |  |
| Lambda-chain reaction | | | | | | | | | | |
| First PCR | | | | | | Second PCR | | | | |
| 15 minutes | | 95 °C | |  | | 15 minutes | 95 °C | |  | |
| 30 seconds | | 94 °C | | 40 x | | 30 seconds | 94 °C | | 30 x | |
| 30 seconds | | 60 °C | |  |  | 30 seconds | 60 °C | |  |  |
| 55 seconds | | 72 °C | |  |  | 45 seconds | 72 °C | |  |  |
| 10 minutes | | 72 °C | |  | | 10 minutes | 72 °C | |  | |
| ∞ | | 4 °C | |  | | ∞ | 4 °C | |  | |
| β-actin (positive control) | | | | | | | | | | |
| 15 minutes | | 95 °C | |  | |  | | | | |
| 60 seconds | | 94 °C | | 45 x | |  |  |  |  |  |
| 60 seconds | | 58 °C | |  |  |  |  |  |  |  |
| 12 seconds | | 72 °C | |  |  |  |  |  |  |  |
| 10 minutes | | 72 °C | |  | |  |  |  |  |  |
| ∞ | | 4 °C | |  | |  |  |  |  |  |
